# Geographical spatial distribution and productivity dynamic change of eucalyptus plantations in China

**DOI:** 10.1038/s41598-021-97089-7

**Published:** 2021-10-05

**Authors:** YuXing Zhang, XueJun Wang

**Affiliations:** 1 Academy of Forest and Grassland Inventory and Planning, National Forestry and Grassland Administration, Beijing, China; 2 Office of the National Forestry and Grassland Administrations Forest Resources Supervision Commissioner in Beijing, Beijing, China

**Keywords:** Environmental impact, Forestry

## Abstract

The *Eucalyptus *spp. is fast-growing and is usually harvested at a young age, which enables efficient and sufficient timber supply. However, its negative impact on soil fertility incurs wide debates. Therefore it is necessary to study on the growing traits of eucalytpus to provide scientific guidance on its plantation management and associated policy-making. In this study, we collected the sample plot data from 9 National Forest Inventories (NFIs) during 1973–2018, China Forest-Land Database Map in 2003 and 2016, as well as climate and elevation data and analyzed how the spatial distribution of eucalyptus plantations in China changes with time. We quantitatively characterized and evaluated the productivity, carbon accumulation capacity, and abandonment rate of eucalyptus plantations. Statistical models on how eucalyptus productivity and abandonment rate change with time are established to evaluate the soil fertility and feasibility for growing eucalyptus plantations and predict the temporal productivity variation. The results show that regions with annual mean temperature of 19–21 °C, annual precipitation of 1400–1600 mm, and elevation of 0–300 m above sea level is most suitable for the growth of eucalyptus. The annual mean productivity of eucalyptus plantations ranges from 4.14–8.57 m^3^ hm^−2^ a^−1^. Higher productivity (9.32–10.88 m^3^ hm^−2^ a^−1^) could be reached in newly cultivated lands. Based on data from the 9th inventory (2014–2018), the mean carbon fixation of eucalyptus is 5.29 t hm^−2^ a^−1^, which is 2.95 and 2.18 times greater than *Pinus massoniana *Lamb. and *Cunninghamia lanceolata *Lamb. Its plantations area accounts for 6.85% of total plantations in China, but it contributes to more than 17.96% of total annual cut from plantations. In Guangdong and Guangxi provinces, areas of eucalyptus plantations are 30.32% and 34.91% of the total plantation area in each province respectively, but eucalyptus plantations contribute to 66.29% and 49.97% of harvested timber stock volume Eucalyptus pla consumes soil fertility significantly. The cumulative abandonment rate (based on area) is about 25%, 50%, and 75% after 5, 10, and 20 years of growing eucalyptus, respectively. The soil fertility decreases significantly after 50 years of growing eucalyptus continuously. In such case, it is difficult to restore the soil fertility. It is suggested that with improved management measures such as proper crop rotation rotating crops properly, it is possible for the abandoned plantations to be reused for growing eucalyptus. Currently the rates of replanting eucalyptus are still below 20% and 30% after 20 and 50 years of without growing eucalyptus, respectively. Although the proportion of eucalyptus area replanted to its abandoned area is now less than 20% in 20 years and less than 30% in 50 years, there is potential to keep increasing the replanting rate. We argue that developing eucalyptus plantations could contribute to global timber supply, help to protect natural forests, increase global carbon storage and fixation, and help to slow down global warming. In conclusion, we should not stop growing eucalyptus despite its high consumption of soil fertility.

## Introduction

*Eucalyptus *spp. is one of the most widely planted broad-leaf forest species in the world. As many as 95 countries have eucalyptus planted in plantations, and the total plantation area has exceeded 22.57 million hectares worldwide. *Eucalyptus *spp. is a fast-growing and high-yielding species characterized by high plant resistance, high tolerance to infertile soil, and ideal trunk shape for timber production. These features make it suitable for a wide variety of uses. *Eucalyptus *spp. has been widely used for large-scale afforestation in many countries, and is one of the four main fast-growing tree species in the world^1^. Eucalyptus together with *Pinaceae* contributes to about 30% of total plantation forests around the globe^2^. In afforestation, commonly-used *Eucalyptus *spp. species include *Eucalyptus urophylla *S.T. Blake, *Eucalyptus grandis *Hill, and *Eucalyptus *dunnii Maiden^3,4^. In 1990, the total area of eucalyptus plantations was 13.414 million hectares around the globe, and the value increased to 14.619 million hectares in 1995^5^. There were 95 countries having eucalyptus planted in 2015, making it the most widely planted broad-leaved forest species in the world^6^. It is estimated that the total area of eucalyptus plantations around the globe has exceeded 22.57 million hectares based on a survey of 65 countries with total eucalyptus plantation area greater than 5000 hectares^7^. Eucalyptus plantations have been developing fast in China since the mid-1990s, and have been ranked first worldwide in terms of growth rate. With massive eucalyptus planting and growing, its contributions to the society as well as impact on regional ecosystem have gained attention from the public, and debates about its disadvantages also exist^7–15^.

Short-term continuous monitoring and experiments of eucalyptus plantations on a regional scale have been done in previous studies^16–23^. With the creation of eucalyptus plantations on a large scale, there is growing concern about its social and ecological benefits, but none of them have been concluded by long-term convincing studies^24^. It is therefore significant to use long-term monitoring data to scientifically assess the impacts of eucalyptus and provide guidance on policy and management measures for planting eucalyptus in countries around the world. China has established a national forest resources inventory system since the 1970s, with surveys every 5 years, which include the productivity of sample forests. Studies focusing on production and temporal distribution of eucalyptus plantations in China based on long-term and continuous surveys of fixed sample plots are limited^25–27^. In this study, we use the continuously monitored data of eucalptus plantation sample plots in China from the 1st to 9th National Forest Inventories (NFIs) and local climate and elevation data to report and calculate the change in spatial distribution, area, stock volume, and carbon storage and fixation of eucalyptus plantations in China. Our work correlates the spatial distribution of eucalyptus plantations with factors such as precipitation, temperature, and elevation, showing the pattern of eucalyptus growth in China. Based on the 5th to 9th NFIs covering a time span of 25 years, we analyze the temporal productivity variation of the sample plots. Our work provides important, previously unavailable data and inferences on growth traits of eucalyptus, which provides objective basis for more scientific and more sustainable management and regulations of eucalyptus plantations.

## Materials, methods and statistical analysis

### Datasets

The data used in this study includes nine NFIs (1st-9th), digital elevation models (DEM; spatial resolution: 30 m), and climatic data (e.g. mean annual temperature, annual precipitation), which ranges over the eucalyptus-planted provinces of southern China. The NFI is done every 5 years, and focuses on all tree species in each of the 415 thousand sample plots (square in shape, 0.0666 hectares). Data from the NFIs includes sample plots index and coordinates, average tree age, mean diameter of tree trunk, average tree height, canopy density and standing forest stock volume as well as other 41 variables. Data on the sampled tree species within the plots are also included, such as trunk diameter (DBH) and stock volume for each tree species (a total of 11 variables). The data of DEM is derived from the ASTER Global Digital Elevation Model (ASTER GDEM), a global digital elevation data product jointly published by NASA and the Japanese Ministry of Economy, Trade and Industry (METI). We also use climate data of 11 provinces in southern China (e.g., Zhejiang, Fujian, Hunan, Jiangxi, Sichuan, Chongqing, Guangxi, Guangdong, Hainan) during 1981–2016 (downloaded from the National Meteorological Information Center http://data.cma.cn; resolution: 0.5° × 0.5°) in this work.

### Data processing

#### Annual mean productivity of sample plots

Datasets of eucalyptus plots in the 5th–9th NFIs are extracted from the complete dataset and turned to spatial vector points. The annual mean production (*P*; m^3^ hm^−2^ a^−1^) of eucalyptus plots is calculated as:

1 $$P = \frac{V}{T}$$

where *V* is the stock volume per unit area (m^3^ hm^−2^), and *T* is the averaged stand age, *V* and *T* are obtained from NFI’S data.

#### Sample plot climate and DEM data

Spatial location, DEM, and annual mean temperature and precipitation at the sampled plots are first projected into the same coordinate system (Albers longitude and latitude project and Krasovsky spheroid; central longitude as 105°E and original latitude as 0°). We use interpolation to get the temperature, precipitation, and DEM data at the sampled plots.

### Spatial distribution analysis based on China Forest-Land Database Map (CFLDM)

China Forest-Land Database Map (CFLDM) is made based on techniques such as remote sensing image analysis, field investigation and validation, forest land property investigation, and GIS data classification. It is a multi-source database which delineates the location and distribution of plantations and natural forests in China, and records their properties (e.g. species, age, area, stock volume) as spatial vectors. Here, we focus on properties of eucalyptus plantations and their extents in 2003 and 2016 (second investigation in that year) from the CFLDM. These 2 years are chosen as the total area of eucalyptus plantations increased significantly from 1999–2003 to 2014–2018. We implemented spatial analysis to analyze and compare the temporal and spatial distribution of eucalyptus plantations in 11 provinces (e.g. Zhejiang, Fujian, Guangdong, Guangxi) in Southern China.

### Calculation of carbon density and storage

We used volume-biomass model to estimate the biomass of sample plots. The biomass per unit area (B; t hm^−2^) is calculated as:

2 $$B = V \times BEF,$$

where V is the stock volume per unit area (m^3^ hm^−2^), and BEF (Biomass Expansion Factor), a unitless factor, is calculated as:

3 $$BEF = \frac{{\mathop \sum \nolimits_{i = 1}^{n} V0 \times \frac{{\mathop \sum \nolimits_{j = 1}^{m} B1}}{{\mathop \sum \nolimits_{j = 1}^{m} V1}}}}{{\mathop \sum \nolimits_{i = 1}^{n} V0}}$$

where V0 is survey measured stock volume in sample plots, V1 is sample plot Diameter at Breast Height (DBH) Class modeled volume, B1 is sample plot DBH Class modeled biomass, *m* and *n* are the numbers of diameter class in the sample plot and the numbers of sample plots, respectively.

We use the *Eucalyptus* model in Feng (1999)^28^, written as:

4 $$W_{S} = 0.0902526\;{\text{D}}^{2.44815}$$

5 $$W_{B} = \, 0.0049163\;{\text{D}}^{2.81779}$$

6 $$W_{L} = 0.012694\;{\text{D}}^{2.26839}$$

7 $$W_{R} = W_{S} + W_{B} + W_{L}$$

8 $$W_{R} = \frac{{W_{T} }}{7.45}$$

where $W_{s}$, $W_{P}$, $W_{B}$, and $W_{L}$ are trunk, bark, twig, and leaf biomasses. $W_{T}$ and $W_{R}$ stand for total biomass above and below the ground. D is DBH.

Based on eucalyptus coverage area and stock volume from the inventories, we calculated the carbon density ($C_{\rho }$; t hm^−2^) and storage ($C_{0}$; t) of eucalyptus plantations:

9 $$C_{\rho } = B \times C_{c}$$

10 $$C_{0} = C_{\rho } \times S$$

where $C_{c}$ and $S$ are carbon coefficient and eucalyptus plantation area (hectares), respectively. $C_{c}$ is a unit-less coefficient, and we use the value of 0.5.

## Results

### Temporal variation and dynamic analysis of eucalyptus forests

Data from the 1st-9th NFIs suggested that the total area of eucalyptus plantations had started to increase since 1973 (Table 1). In 1973–1976, Eucalyptus plantations only existed in Guangxi and Guangdong (including Hainan) in China with a total area of 23.0 × 10^4^ hectares, taking up 0.38% of total forest area in China. The stock volume was 372.0 × 10^4^ m^3^, about 0.04% of that in China. In 2014–2018, the eucalyptus plantation area increased to 546.74 × 10^4^ hectares, about 24 times of that in 1973–1976. The growing stock has increased to 21,562.90 × 10^4^ m^3^ in 2014–2018 which increased about 58 times from 1973–1976.

**Table 1:** Eucalyptus plantation area and stand volume in different time periods by province.

Province	1973–1976		1977–1981		1984–1988		1989–1993		1994–1998		1999–2003		2004–2008		2009–2013		2014–2018	
	A	V	A	V	A	V	A	V	A	V	A	V	A	V	A	V	A	V
Zhejiang	–	–	–	–	–	–	–	–	–	–	–	–	–	–			0.24	0.60
Fujian	–	–	–	–	–	–	–	–	0.96	19.27	1.68	23.12	15.87	444.64	27.14	2040.65	20.88	1347.94
Jiangxi	–	–	–	–	–	–	–	–	–	–	–	–	1.28	0	4.49	54.49	1.28	11.67
Hunan	–	–	–	–	–	–	–	–	–	–	–	–	1.60	0	2.25	8.36	1.92	33.92
Guangdong	18	290	16.52	590.36	17.27	292.98	18.23	422.68	29.27	460.82	40.77	912.44	132.41	2119.9	171.27	5177.83	186.65	4946.27
Guangxi	5	82	4.8	42.65			5.76	66.71	7.20	290.07	14.88	216.49	53.36	1001.37	165.24	6049.76	256.05	10,989.47
Hainan	–	–	–	–	10.8	109.14	15.96	298.1	17.03	504.43	16.67	538.92	19.3	507.38	14.62	524.9	12.94	555.60
Chongqing	–	–	–	–	–	–	–	–	–	–	–	–	0.48	22.24	4.65	79.07	3.54	129.97
Sichuan	–	–	–	–	0.64	7.35	1.28	22.48	–	–	2.43	72.22	6.32	127.77	16.02	408.2	18.43	1075.86
Guizhou	–	–	–	–	–	–	–	–	–	–	–	–	–	–	0.96	10.42	2.57	59.71
Yunnan	–	–	–	–	0.48	0	1.92	5.61	6.24	112.49	5.76	171.54	24	354.59	38.88	1682.49	42.24	2411.89
Total	23	372	21.32	633.01	29.19	409.47	43.15	815.58	60.70	1387.08	82.19	1934.73	254.62	4577.89	445.52	16,036.17	546.74	21,562.90

The letters “A” and “V” refer to cover area (unit: 10^4^ hm^2^) and stand volume (unit: 10^4^ m^3^), respectively.

The stock volume per unit area of eucalyptus plantations did not increase significantly from 1973–2008, ranging from 14–30 m^3^/hectares, but it increased rapidly from 2009 to 2018, reaching 39.43 m^3^/hectares. This increase occurred because China started to focus and value the development of eucalyptus plantations. As a. result, plantations expanded rapidly, and the need for eucalyptus with greater trunk radius increased. The extended harvest cycle of eucalyptus plantations, not the rise of eucalyptus productivity, caused the increase in stock volume per unit area^29–31^.

**Figure 1:**
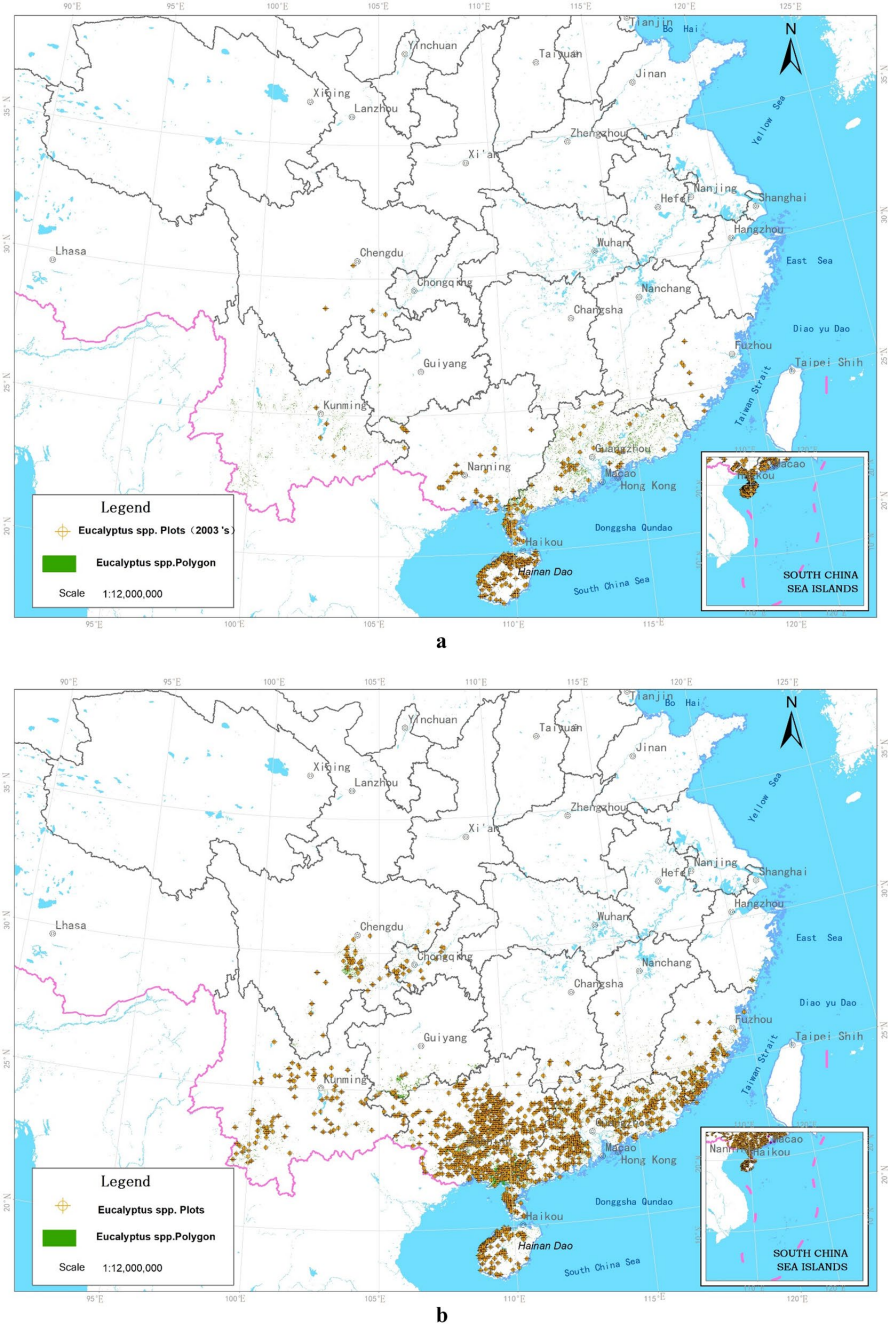
Distribution of eucalyptus in the south of China [(**a**) 2003; (**b**) 2016]. This figure was created by spatially overlaying spatial sample plots data from National Forest Inventory (NFI) and patch vectors data from China Forest-Land Database Map (CFLDM), (**a**) shows that the point data are from 6th NFIs eucalyptus sample plots and the polygon vector data are from the 2003 CFLDM; (**b**) shows that the point data are from 9th NFIs eucalyptus sample plots and the polygon vector data are from the 2016 CFLDM. The extents of eucalyptus plantations is mainly concerned with 11 provinces (e.g. Zhejiang, Fujian, Guangdong, Guangxi) in Southern China.

Based on CFLDM, the distribution of eucalyptus plantations in 2003 and 2016 are mapped (Fig. 1a,b). It suggests that the distribution of eucalyptus plantations extended from Leizhou Peninsula, Guangdong and Hainan Province to the north (Guangxi, Hunan, and Guizhou provinces), east (Fujian and Jiangxi provinces), and west (Yunan and Sichuan provinces). This is consistent with data from the NFIs. The widespread expansion of eucalyptus also leads to several regions with clustered plantations.

### Changes in spatial distribution

Based on the database of sample plots (including climate and elevation data) and sampled eucalyptus, we analyze the distribution of eucalyptus plantations*,* and how it is affected by elevation and climate conditions. It is found that most eucalyptus plantations are within the region of 110°23′–120°5′E and 18°21′–30°39′N. The annual mean temperature within this region ranges from 11 to 25 °C with an average of 19.5 °C, and the annual precipitation ranges from 600 to 2000 mm with an average of 1455 mm. Elevation in this region is 0–2500 m with an average of 338 m.

To find out the most suitable conditions for eucalyptus growth and its plantation management, we classify this region based on their elevation and climate conditions. The classification is done separately and independently for each factor (i.e., elevation, temperature, and precipitation). In terms of elevation, the region is assigned to seven grades from below 300 m to 2100 m with an interval of 300 m in between (i.e., below 300 m, 300–600 m, 600–900 m, 900–1200 m, 1200–1500 m, 1500–1800 m, 1800–2100 m). Land with elevation above 2100 mm has limited eucalyptus plantations, and thus is not taken into consideration. Similar criterion is applied to the classification based on annual mean temperature and annual precipitation. The grades are from 11 to 25 °C within an interval of 2 °C for temperature and 600–2000 mm with an interval of 200 mm for precipitation.

**Figure 2:**
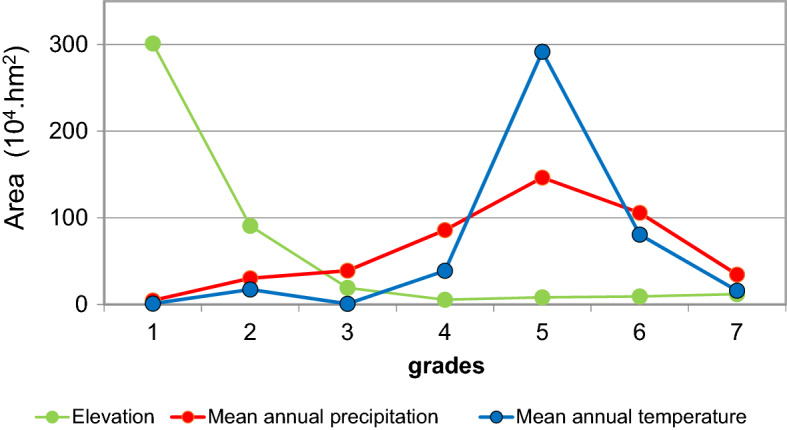
Area of eucalyptus plantations in China based on grades defined in text.

We examine how the eucalyptus forest area changes with these factors. This is done by plotting the eucalyptus plantation area within a certain group against the corresponding grade number (Fig. 2). Eucalyptus is mostly distributed below 300 m, reaching an area of 301.1 × 10^4^ hectares and counting for 67.58% of the total eucalyptus plantation area in China. Eucalyptus occurs rarely in areas with elevation above 900 m.

Eucalyptus is sensitive to temperature, and its distribution is limited within areas with annual mean temperature below 19 °C. Eucalyptus is mostly distributed within areas with annual mean temperature of 19–21 °C. Areas with annual mean temperature within this range have a total of 291.49 × 10^4^ hectares eucalyptus plantations, approximately 65.43% of all eucalyptus plantations in China. This result is slightly different from previous studies^3,5^, which suggests that eucalyptus prefers areas with mean annual temperature above 20 °C.

Eucalyptus has a high tolerance to annual precipitation. Eucalyptus plantations can be found in areas with annual precipitation ranging from 600–2000 mm. It should be noted that this is related to irrigation conditions in production and management. However, in areas with annual precipitation below 600 mm, Eucalyptus plantations are extremely rare. Areas with annual precipitation of 1400–1600 mm (and without considering other factors) have the largest portion of eucalyptus plantations, whose total area reaches 146.49 × 10^4^ hectares. This accounts for about 32.94% of total eucalyptus plantations in China.

### Productivity analysis of eucalyptus

#### Variability in mean productivity

**Figure 3:**
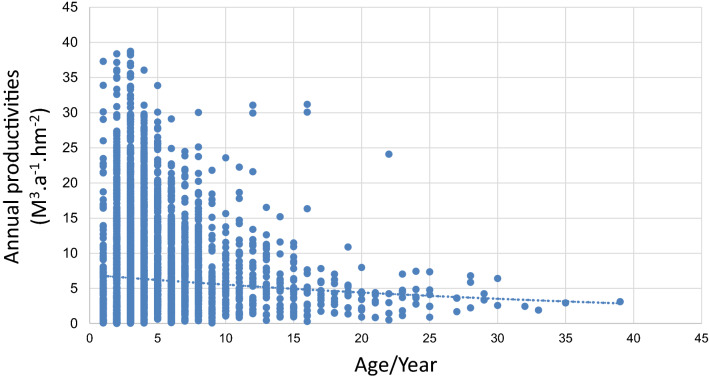
Relationship between age and productivity of eucalyptus sample plots.

Eucalyptus annual productivity for each province based on the 5th to 9th NFIs (no digitized data for the 1th to 4th NFIs) is calculated, which includes 3564 sample plots (Table 2). Among which, 769 sample plots had been harvested at the time of the survey, and to remove the influence of these 769 sample plots, their data were removed during the productivity of the eucalyptus age-productivity relationship graph (see Fig. 3), which shows that the period of maximum productivity for eucalyptus lasts for approximately 2–3 years. Its productivity declines rapidly after 10 years of growth. Therefore, the harvest cycle of eucalyptus is normally 4–5 years. After coppicing and growing for another 4–5 years, Eucalyptus will be harvested again, which will be followed by its replanting.

**Table 2:** Basic sample plot statistics (quantity, mean and maximum annual productivity) of eucalyptus plantations by province from the 5th to 9th NFIs.

Province	5th (1994–1998)			6th (1999–2003)			7th (2004–2008)			8th (2009–2013)			9th (2014–2018)		
	NO	M	MAX	NO	M	MAX	NO	M	MAX	NO	M	MAX	NO	M	MAX
Zhejiang	–	–	–	–	–	–	–	–	–	–	–	–	1	0.36	0.36
Fujian	4	2.81	6.96	7	3.95	10.72	66	7.82	33.32	113	13.75	36.69	87	9.48	29.54
Jiangxi	–	–	–	–	–	–	–	–	–	7	1.79	8.15	2	1.91	3.53
Hunan	–	–	–	–	–	–	–	–	–	7	1.2	3.44	6	2.70	7.96
Guangdong	61	4.53	24.49	85	5.38	34.91	276	5.38	37.15	357	6.8	38.38	389	6.27	33.34
Guangxi	15	5.43	10.23	31	6.29	19.37	111	4.04	24.12	344	9.76	38.51	533	11.39	38.73
Hainan	142	3.88	18.72	139	4.21	22.26	161	4.64	24.5	122	4.7	14.15	108	6.65	29.03
Chongqing	–	–	–	–	–	–	3	2.33	3.41	29	0	0	22	4.25	9.35
Sichuan	–	–	–	4	2.5	4.97	13	2.65	7.86	33	4.29	14.5	38	7.08	17.42
Guizhou	–	–	–	–	–	–	–	–	–	3	2.88	7.66	8	4.99	10.63
Yunnan	4	3.86	6.5	6	6.43	24.17	50	2.99	14.89	81	6.50	27.88	88	6.55	32.01
Total	226	4.14	24.49	273	5.72	34.91	687	4.94	37.15	1096	7.86	38.51	1282	8.57	38.73

“NO.” refers to the amount of sample plots; “M” and “MAX” refer to mean and maximum annual productivity, respectively. (unit: m^3^ hm^−2^ a^−1^). The same below.

#### Variability in *eucalyptus* productivity

Eucalyptus productivity for each province based on the 5th to 9th NFIs is calculated and shown in Table 2. It can be seen that from 1994 to 2018, mean and maximum productivities of eucalyptus plantations have increased. This is especially show for Guangxi and Fujian Provinces during 2009–2018. The averaged productivity of eucalyptus plantations in China increased from 4.14 to 8.57 m^3^ hm^−2^ a^−1^ from 1994–1998 to 2014–2018, which can be explained by the improved management of eucalyptus plantations (e.g., high soil fertility for newly cultivated lands and improved ability for irrigation) and their expansion.

Data from the 5th to 9th NFIs suggest that a lot of sampled plots were no longer used for growing eucalyptus before the next inventory (Table 3). There were 226, 273, 687, and 109 eucalyptus plots in the 5th, 6th, 7th, 8th inventories, respectively, and in the corresponding next NFI (6th, 7th, 8th, 9th), only 150, 179, 544, and 848 of these sample plots were left unabandoned. This suggests that 33.63%, 34.43%, 20.82%, and 22.63% of the plots were abandoned before the next inventory. New plots have been included in each inventory, but large portions of these plots were abandoned as well. There are 123, 508, and 552 new eucclyptus plots in the 6th, 7th, 8th inventories, and 76, 413, and 433 of them were left unabandoned in the next inventories. The land abandonment rates for them are 38.21%, 18.70%, 21.56%, respectively.

**Table 3:** Quantity of newly-cultivated, retained, and abandoned sample plots during different NFIs.

	Plot type	5th (1994–1998)		6th (1999–2003)			7th (2004–2008)			8th (2009–2013)			9th (2014–2018)		
		NO	M	NO	M	Q	NO	M	Q	NO	M	Q	NO	M	Q
5th	Retained plot	226	4.14	150	6.03	76	103	5.71	47	72	6.00	31	55	6.03	17
6th	Newly-cultivated			123	5.34		76	6.13	47	59	9.30	17	38	5.64	21
7th	Newly-cultivated						508	4.60		413	7.59	95	322	7.70	91
8th	Newly-cultivated									552	8.15		433	9.24	119
9th	Newly-cultivated												434	9.13	
Total		226	4.14	273	5.72	76	687	4.94	96	1096	7.86	143	1282	8.57	248

“Q.” refer to abandoned sample plots.

We examine how the productivity changes with time for eucalyptus plantations that have been operated for more than 20 years. From the 5th to 9th NFIs, we find 55 and 38 such (operating for more than 25 and 20 years, respectively; Table 4). It is found that the productivity of eucalyptus is relatively low in the first 5 years of its growing. The productivity increases in the 5th–10th years, and reaches its peak after 10–15 years of growing eucalyptus. For land that have been continuously growing eucalytptus for 15–25 years or more, the productivity decreases significantly. This is due to the decrease in soil fertility.

**Table 4:** Relationship between Continuous planting time and Mean productivity of reserved and newly-cultivated eucalyptus sample plots during different NFIs (unit: m^3^ hm^−2^ a^−1^).

Continuous planting time (a)	Mean productivity in 5th	Number of plots	Mean productivity in 6th	Number of plots	Mean productivity	Number of plots
1–5	4.14	226	5.34	123	4.56	349
6–10	6.03	150	6.13	76	6.06	226
11–15	5.71	103	9.30	59	7.02	162
16–20	6.00	72	5.64	38	5.88	110
20–25	6.03	55			6.03	55

**Figure 4:**
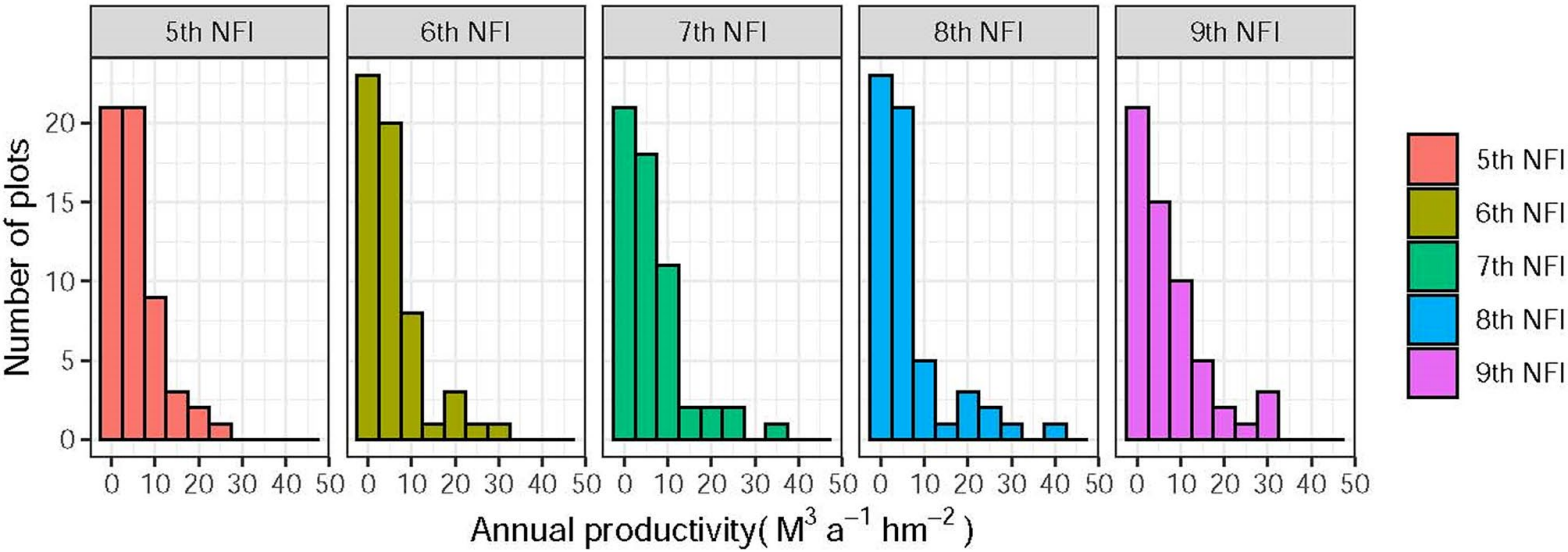
Distribution of mean annual productivity for sample plot from different NFIs.

**Figure 5:**
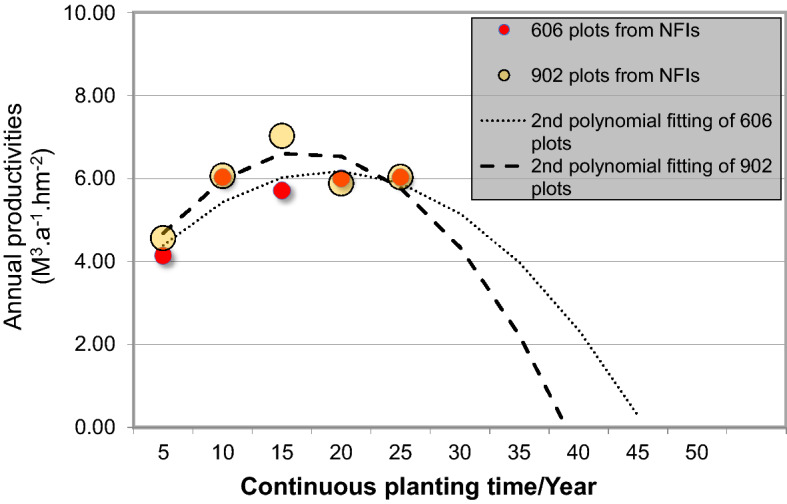
Statistical model showing how mean annual productivity of eucalyptus sample plots changes with time.

Most (~ 90%) of the sample plots have mean annual productivity below 10 m^3^ hm^−2^ a^−1^ (Fig. 4). The productivity reaches its peak after 10–15 years of growing eucalyptus (mean annual average: 7.0175 m^3^ hm^−2^ a^−1^), and starts to decrease afterwards. Statistical model (Table 5) is established between productivity and age of eucalyptus plots. The results suggest that eucalyptus productivity follows a consistent pattern: it increases with time until a peak and then decrease (Fig. 5), and this applies to the old, newly-included plots and their average. The statistical model also agrees to the observed data, suggesting that the productivity peak is reached 10–15 years after the planting of eucalyptus, and the productivity reaches its minimum or even zero after 50 years of growing eucalyptus continuously.

**Table 5:** Productivity prediction model of multi-stage reserved and increase eucalyptus sample plots.

Data	Reliability-calculating model	
	Model	R^2^
Modeling of 606 plots	y = − 0.2221X^2^ + 1.7079X + 2.902	0.7849
Modeling of 902 plots	y = − 0.3420X^2^ + 2.3268X + 2.6919	0.7765

#### Soil fertility variation of eucalyptus plantations

How eucalyptus affects soil fertility is not well-studied. Here, based on 948 sample points from Tang^32^, which includes monitoring of soil fertility of eucalyptus plantations from 1993 to 2018, we report and study the temporal soil fertility variation for eucalyptus plantations. After 25 years of growing eucalyptus, acidification of the corresponding lands persists. The pH value changed to 4.63 in 2018, a 4.14% decrease compared to that in1993. The organic content within the soil reached its minimum of 17.98 g/kg in 2018, a decrease of 23.19% compared to 1993. Total nitrogen content of the investigated samples changed from 2.11 to 1.98 g/kg, and total phosphorus content decreased from 1.12 to 0.75 g/kg. The temporal variation of potassium does not change in a consistent pattern with time. Alkaline hydrolysis of nitrogen and available potassium content in 2018 are significantly lower than those in 1993. From more to less, the rank of soil fertility indicator affiliation polygon area is 1993 > 1998 > 2003 > 2013 > 2018 > 2008. The rank of soil fertility index is 1993 > 1998 > 2018 > 2003 > 2013 > 2008. It decreased first, and then increased. The minimum soil fertility (0.475) was reached in 2008 (22.51), which is smaller than that in 1993. The soil fertility decreases at the greatest rate after 15 years of growing eucalyptus. This argument from Tang^32^ is consistent with this work (Table 6). Soil fertility generally decreases with the age of eucalyptus plantations.

**Table 6:** Evolutionary characteristics of soil chemical indicators in eucalyptus plantation forests.

Indicators	Items	1993	1998	2003	2008	2013	2018	CV /%
pH	Maximum	8.67	8.05	8.54	8.54	7.95	6.71	
	Minimal	3.32	3.95	3.99	3.24	3.49	4.29	
	Mean	4.83	4.77	4.83	4.48	4.57	4.63	16.54
	Standard deviation	1.21	0.64	0.79	1.01	0.65	0.35	
Organic matter /(g/kg)	Maximum	43.22	38.73	44.09	38.84	39.52	39.27	
	Minimal	4.68	7.39	1.95	3.51	1.27	5.61	
	Mean	23.41	28.32	26.23	29.22	25.23	17.98	49.56
	Standard deviation	11.13	12.63	13.61	17.83	16.07	7.71	
Total nitrogen (g/kg)	Maximum	4.35	2.54	3.83	4.76	4.07	4.83	
	Minimal	0.21	0.17	0.34	0.22	0.04	0.38	
	Mean	2.11	1.12	1.27	1.37	1.57	1.98	44.82
	Standard deviation	0.46	0.51	0.56	0.95	0.98	0.33	
Total phosphorus (g/kg)	Maximum	2.77	1.84	2.21	1.49	2.41	2.12	
	Minimal	0.14	0.25	0.29	0.11	0.06	0.12	
	Mean	1.12	1.16	1.41	0.71	0.83	0.75	64.47
	Standard deviation	0.67	0.93	0.84	0.47	0.79	0.16	
Total potassium (g/kg)	Maximum	33.43	27.76	21.98	26.81	27.54	32.58	
	Minimal	1.04	1.59	1.27	2.52	2.81	1.65	
	Mean	7.73	14.85	11.59	15.52	15.27	9.27	63.22
	Standard deviation	4.33	7.71	5.89	9.85	11.03	7.87	
Alkaline nitrogen (mg/kg)	Maximum	167	183.29	175.61	131.3	121.2	155.76	
	Minimal	29	19.41	23.4	16.31	17.71	24.1	
	Mean	106.29	113.86	132.61	78.21	91.86	78.25	52.91
	Standard deviation	44.43	58.26	78.72	50.76	65.18	22.93	
Active phosphorus (mg/kg)	Maximum	7.21	9.33	8.9	5.11	7.41	10.81	
	Minimal	0.23	0.11	0.12	0.21	0.11	0.61	
	Mean	2.99	5.28	2.13	2.77	3.67	5.62	28.49
	Standard deviation	1.03	0.53	0.72	0.26	0.75	1.93	
Fast-acting potassium (mg/kg)	Maximum	151.39	148.61	100.41	108.88	116.8	126.05	
	Minimal	7.13	5.6	6.83	12.19	3.33	5.31	
	Mean	123.29	46.83	29.74	40.61	42.61	56.62	32.11
	Standard deviation	24.43	13.47	9.78	18.32	11.59	22.04	

“CV” refer to Coefficient of variation.

In addition, Parfitt et al.^33^ studied the variation of soil fertility of pine plantations in New Zealand for a period of 20 years, and found that long-term successive rotations lead to an increase of the soil C/N ratio. Carbon is lost at a speed much greater than nitrogen. Successive rotations of eucalyptus lead to environmental issues such as decrease in soil fertility and ecological diversity and soil erosion. These would limit the sustainable management of eucalyptus plantations^34–40^.

#### Abandonment of sample plots

We find that many sample plots were not used for growing eucalyptus anymore after each inventory. The abandonment rate is high, ranging from 18.7 to 38.21%. The 226 eucalyptus sample plots in the 5th inventory decreased to 103 (the others are abandoned) during the 7th inventory, and the land abandonment rate was 31.33%. In the 8th and 9th inventories, the abandonment rates are 30.10% and 23.61%, respectively. The cumulative land abandonment rates are 33.63%, 54.43%, 68.15%, and 75.66% after 5, 10, 15, and 20 years of growing eucalyptus, respectively (Table 7).

**Table 7:** Quantity (rates) of retained and abandoned sample plots after certain periods of plantation management.

Original plots	Sample quantity	Abandoned within 5 years		Abandoned within 10 years		Abandoned within 15 years		Abandoned within 20 years	
Type		Retained plots	Cumulative abandonment rate	Retained plots	Cumulative abandonment rate	Retained plots	Cumulative abandonment rate	Retained plots	Cumulative abandonment rate
Original plots (1994–1999)	226	150	33.63	103	54.43	72	68.15	55	75.66
Newly-cultivated (1999–2003)	123	76	38.21	59	52.03	38	69.10		
Newly cultivated (2004–2008)	508	413	18.70	322	36.61				
Newly-cultivated (2009–2013)	552	433	21.56						
Newly-cultivate (2014–2018)	434								
Mean abandoned rate	1843		23.92		24.26		32.10		23.61
Cumulative abandonment rate			23.92		43.52		68.48		75.66

There are a total of 1843 eucalyptus plots from the 5th to 9th NFIs. In the last NFI, there are 1282 sampled plots still growing eucalyptus, and the rest 561 plots are abandoned. The averaged land abandonment rates of these plots every 5 years are 23.92%, 24.26% (43.52% cumulatively), 32.10% (68.48% cumulatively), and 23.61% (75.66% cumulatively) over 5, 10, 15, and 20 years, respectively.

These data suggest that the abandonment rate of eucalyptus plantations reaches its peak (about one third) after 15 years of operation. For other time intervals (i.e., 5, 10, and 20 years), the rate remains at around 25%. This is related to the management of eucalyptus plantations in the south of China: the first eucalyptus harvest cycle is about 6 years. The second generation of eucalyptus reproduces by division propagation (sprout naturally) with 4 years of harvest cycle, and the third generation follows the same pattern. These amount to 15–16 years-long period for plantation management. Eucalyptus requires stubble-cleaning after twice of division propagations (sprout reproduction), and needs to be re-planted. This is consistent with the timing of abandonment rate peak as stated above. It is highly likely that the eucalyptus plantations are abandoned due to the low soil fertility, and plantation managers or land owners decide to stop growing eucalyptus as a result.

**Figure 6:**
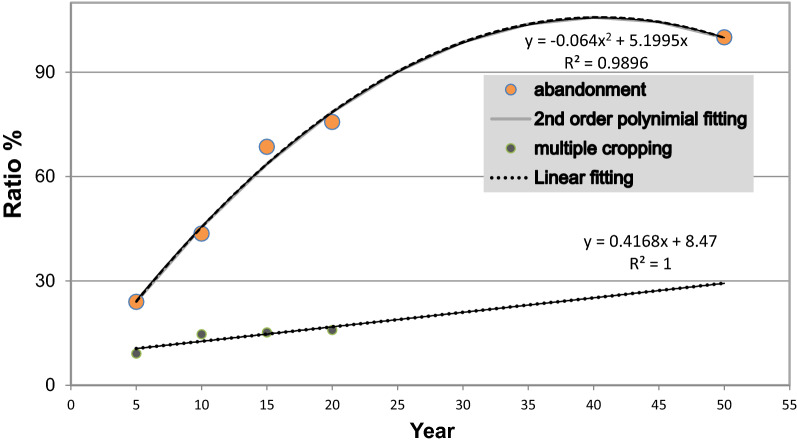
Statistical model showing how abandonment and replanting rates of eucalyptus plantations change with time.

A simple statistical model (second-order polynomial) is established between eucalyptus plantations abandonment rate and time (Fig. 6), which suggests that all plantations will stop growing eucalyptus after 50 years, and the corresponding lands will be used for other purposes. The expansion of eucalyptus plantations relies on sustained cultivation of new lands (land reclamation). The total area of eucalyptus plantations reached 5,647,400 hectares in the 9th NFI, but only 4.29% of them (that) have been continuously growing eucalyptus since the 5th NFL (i.e., 24.34% of the plots from the 5th NFI are kept).

There are two main reasons to explain the loss of eucalyptus plantations. The land might be taken over for non-agricultural use (e.g., infrastructure and building construction), or they could be used for growing other crops. The latter is help for soil fertility restoration and soil microorganism readjustment. As most eucalyptus plantations in China are cultivated on lands with poor growing conditions, most of them were abandoned voluntarily by the land owner or plantation manager as stated earlier.

After harvest, eucalyptus plantations could be reused for the continuation of eucalyptus growing or used for other purposes (e.g., growing other crops). The plots that were temporarily not used for growing eucalyptus could be used for re-growing it under certain conditions after a certain time period. We investigate the replanting rate of the 561 abandoned eucalyptus plantations, and study whether the abandoned plantations are used for growing other outcrops, and, if so, the corresponding tree species (Tables 8, 9, 10, 11). The 6th NFI data suggests that there are 76 plots abandoned after the 5th NFI. Their replanting rates are 2.63%, 7.89% (10.53% cumulatively), 0.00% (10.53% cumulatively), and 5.26% (15.79% cumulatively) within every 5 years, and after 5, 10, and 15 years of abandonment. For all the 561 abandoned plots, replanting rates are 9.09%, 5.53% (cumulatively 14.62% within 10 years), 0.53% (cumulatively 15.15% within 15 years), 5.26% (cumulatively 15.86% within 20 years) within every 5 years, and after 5, 10, and 15 years of abandonment. These suggest that about five sixth of the abandoned plots had not replanted eucalyptus for at least 20 years since abandonment.

**Table 8:** Land use of eucalyptus plantation sample plots during different NFIs.

Abandonment period	Quantity of abandoned plots	6th (1999–2003)			7th (2004–2008)			8th (2009–2013)			9th (2014–2018)		
		Growing eucalyptus	Growing non-eucalyptus species	Non-plantation use	Growing eucalyptus	Growing non-eucalyptus species	Non-plantation use	Growing eucalyptus	Growing non-eucalyptus species	Non-plantation use	Growing eucalyptus	Growing non-eucalyptus species	Non-plantation use
6th	76	2	48	26	8	44	24	8	46	22	12	42	22
7th	47				2	36	9	5	32	10	5	30	12
	47				5	36	6	16	25	6	19	23	5
8th	31							3	23	5	4	22	5
	17							1	12	4	2	9	6
	95							12	69	14	21	60	14
9th	17										1	12	4
	21										1	15	5
	91										11	66	14
	119										13	87	19
Total	561	2	48	26	15	116	39	45	207	61	89	366	106
Percentage	–	2.63	63.16	34.21	8.82	68.24	22.94	14.38	66.13	19.49	15.86	65.24	18.89

For example, in the 6th NFI, 2, 48, and 26 sample plots from the 5th NFI are used for growing eucalyptus, non- eucalyptus species, and non-plantation use, respectively.

**Table 9:** Temporal change of tree species planted in sample plots.

Abandonment period	Abandoned plot	6th (1999–2003)						7th (2004–2008)						8th (2009–2013)						9th (2014–2018)					
	Quantity	A	B		C	D	E	A	B		C	D	E	A	B		C	D	E	A	B		C	D	E
			B1	B2					B1	B2					B1	B2					B1	B2			
6th	76	4	2	4		26	40	4	8	1		31	32	4	8	2		31	31	3	12	1		28	32
7th	47							2	2	8	1	17	17	2	5	5	1	21	13	2	5	5	1	21	13
	47							2	5	15		3	22	1	16	12	1	6	11	2	19	12		4	10
8th	31														3	5	1	13	9		4	5		15	7
	17													2	1	4		2	8	2	2	3		3	7
	95													7	12	17	6	12	41	5	21	18	5	14	32
9th	17																			1	1	5	1	2	7
	21																				1	3	2	2	13
	91																			10	11	22	9	5	34
	119																			11	13	30	12	9	44
Total	561	4	2	4	0	26	40	8	15	24	1	51	71	16	45	45	9	85	113	36	89	104	30	103	199
Percentage	100	5.26	2.63	5.26	0.00	34.21	52.64	4.71	8.82	14.12	0.59	30.00	41.76	5.11	14.38	14.38	2.88	27.16	36.09	6.42	15.86	18.54	5.35	18.36	35.47

*A* conifers, *B* broad-leaf trees, *B1* eucalyptus, *B2* Broad-leaf trees other than eucalyptus, *C* mixed forest, *D* economic tree species, *E* not used for plantation.

**Table 10:** Replanting rate of eucalyptus plantations.

Inventory Index	Total quantity	Replanting within 5 years		Replanting within 10 years		Replanting within 15 years		Replanting within 20 years	
		Quantity	Rate (cumulative in bracket)	Quantity of abandoned plantations	Rate (cumulative in bracket)	Quantity of abandoned plantations	Rate (cumulative in bracket)	Quantity of abandoned plantations	Rate (cumulative in bracket)
6th	76	2	2.63 (–)	6	7.89 (10.53)	0	0 (10.53)	4	5.26 (15.79)
7th	94	7	7.45 (7.45)	14	14.89 (22.34)	3	3.19 (25.53)		
8th	143	16	11.19 (11.19)	11	7.69 (18.88)		0.00 (–)		
9th	248	26	10.48 (10.48)		0.00 (10.48)		0.00 (–)		
Total	561	51	9.09 (9.09)	31	5.53 (14.62)	3	0.53 (15.15)	4	5.26 (15.86)

**Table 11:** Productivity of plantations that have replanted eucalyptus.

Abandonment time period	Quantity of abandoned plantations	Replanting productivity (< 5 years of abandonment)		Replanting productivity (< 10 years of abandonment)		Replanting productivity (< 15 years of abandonment)		Replanting productivity (< 20 years of abandonment)	
		NO	M	NO	M	NO	M	NO	M
1999–2003	76	2	0	6	2.49	0		4	8.93
2004–2008	94	7	0	14	5.90	3	10.88		
2009–2013	143	16	0	11	9.32				
2014–2018	248	26	0						
Total	561	51	0	31	6.45	3	10.88	4	8.93

A simple statistical model is established between replanting rate and time (second-order polynomial) based on the current data (Fig. 5). It suggests that the replanting rates after 30 and 50 years are around 20% and 30%, respectively. These suggest that if the plantation management does not improve significantly, it would be difficult to maintain the current supply of eucalyptus and areal distribution of its plantations in the long term. It is necessary to rely on both land rotation and cultivation of new lands to maintain the current supply of eucalyptus.

The NFI data suggests that very few eucalyputs plots are turned to non-plantation purposes. The exception is from the 6th NFI in which 34.21% of plots have been used for other purposes after harvest. This rate is below 20% for all other inventory data. A lot of abandoned eucalyptus plantations are still used as plantations, and they are for growing eucalyptus, and the rate of regrowing eucalyptus tends to remain low for a long period of time (below one sixth after 20 years based on current data). This is because eucalyptus grows fast with high productivity, and it has high demand for soil fertility and water. Land rotation is necessary after a few harvest cycles to restore the soil fertility, which would take relatively long period of time before the land becomes suitable to regrow euccalyptus. Among the 561 abandoned eucalyptus plots, broad-leaf and economic tree species are the most commonly planted species after stop growing eucalyptus (e.g., rubber tree and Lychee; 18.54% and 18.36%; Table 9). The greater variability of land use for the abandoned plots suggests greater management intensity. Afforestation with eucalyptus is dominated by short rotation period (harvest cycle). Frequently modifying tree species planted within plantations helps maintain a high productivity of the land.

### Carbon storage and fixation of eucalyptus plantations

#### Variability in carbon storage

Based on the 9th NFI data and Eqs. (2–8), we calculate the BEF of eucalyptus in each province (Table 12). The results suggest that the BEF ranges from 0.982–1.652 with a weighted average of 1.236 (weight determined by stock volume).

**Table 12:** BEF of eucalyptus by province.

Province	BEF above the ground	BEF under the ground	BEF
Zhejiang	1.6752	0.2248	1.9000
Fujian	0.8658	0.1162	0.9820
Jiangxi	1.4566	0.1955	1.6521
Hunan	1.4109	0.1893	1.6002
Guangdong	1.1604	0.1558	1.3162
Guangxi	1.0542	0.1415	1.1957
Hainan	1.2183	0.1635	1.3818
Chongqing	1.1676	0.1567	1.3243
Sichuan	1.1078	0.1487	1.2565
Guizhou	1.4317	0.1921	1.6238
Yunnan	1.1562	0.1551	1.3113
Weighted average	1.0876	0.1460	1.2336

Calculation from Eqs. (9) and (10) suggests that the total carbon storage (excluding harvest volume) of eucalyptus in China is 2.40 TgC (1973–1976, 1Tg = 10^12^ g), 4.14 TgC (1977–1981), 2.73 TgC (1984–1988), 5.42 TgC (1989–1993), 9.73 TgC (1994–1998), 12.58 TgC (1999–2003), 28.90 TgC (2004–2008), 98.61 TgC (2009–2013), and 133.00 TgC (2014–2018) in different time periods in the past 45 years (Table 13).

**Table 13:** Eucalyptus carbon density and storage by province.

	1973–1976		1977–1981		1984–1988		1989–1993		1994–1998		1999–2003		2004–2008		2009–2013		2014–2018	
Province	C_p_	C_0_	C_p_	C_0_	C_p_	C_0_	C_p_	C_0_	C_p_	C_0_	C_p_	C_0_	C_p_	C_0_	C_p_	C_0_	C_p_	C_0_
Zhejiang																	2.08	0.01
Fujian													6.87	2.18	18.46	10.02	15.85	6.62
Jiangxi															5.01	0.45	3.91	0.10
Hunan															1.56	0.07	7.03	0.27
Guangdong	5.31	1.91	11.77	3.89	5.59	1.93	7.62	2.78	5.18	3.03	7.36	6.00	5.27	13.95	9.95	34.08	8.72	32.55
Guangxi	4.90	0.49	2.60	0.25			3.47	0.40	12.01	1.73	4.33	1.29	5.61	5.99	10.94	36.17	12.83	65.70
Hainan					3.47	0.75	6.45	2.06	10.25	3.49	11.16	3.72	9.09	3.51	12.41	3.63	14.84	3.84
Chongqing													15.63	0.15	5.59	0.52	12.15	0.86
Sichuan					3.91	0.05	5.47	0.14			9.26	0.45	6.33	0.80	7.99	2.56	18.34	6.76
Guizhou															4.17	0.08	9.34	0.48
Yunnan								0.04	11.86	1.48	9.72	1.12	4.83	2.32	14.18	11.03	18.71	15.81
Total	5.22	2.40	9.71	4.14	4.68	2.73	6.28	5.42	8.01	9.73	7.65	12.58	5.68	28.90	11.07	98.61	12.16	133.00

“Cp” and “C_0_” refer to Forest biomass carbon density (unit: t^4^ hm^−2^) and Biomass carbon stocks (unit: TgC), respectively.

The carbon storage of eucalyptus increased rapidly in the past 45 years especially since the end of last century. This is due to the rapid expansion of eucalyptus plantations in China, and its carbon storage in 2014–2018 is 55.42 times of that in 1973–1976. The carbon density per square hectometer also increases from 5.22 MgC (1 Mg = 10^6^ g) in 1973–1976 to 12.16 MgC in 2014–2018, about 2.33 times of the former.

#### Carbon fixation

The mean annual productivity of eucalyptus is 8.57 m^3^ hm^−2^ a^−1^ in 2014–2018 based on the 9th NFI. This is a lot greater compared to other species widely planted in the same areas (*Pinus massoniana *Lamb.: 2.91 m^3^ hm^−2^ a^−1^; *Cunninghamia lanceolata* Lamb.: 3.93 m^3^ hm^−2^ a^−1^). Using the stock volume biomass method with BEF being 1.2336 (from previous calculation) and carbon storage coefficient of 0.5, the mean annual carbon fixed by eucalyptus is 5.29 t hm^−2^ a^−1^, which are about 2.95 and 2.18 times that of *Pinus massoniana *Lamb. (1.79 t hm^−2^ a^−1^) and *Cunninghamia lanceolata* Lamb. (2.42 t hm^−2^ a^−1^), respectively. This shows that eucalyptus is characterized by high biomass productivity and high carbon fixation capability. It thus plays an important role in maintaining the carbon balance in China.

## Discussion

Eucalyptus prefers warm temperature, and has low cold tolerance. Based on the surveyed sample plots, the northern limit of its plantations is 31°3′N, 107°33′E with an elevation of 410 m a.s.l. and annual mean temperature of 12 °C. Their western limit is 22°18′N, 99°25′E with an elevation of 1380 m a.s.l. and annual mean temperature of 17 °C. Their eastern limit is 27°40′N, 120°27′E with an elevation of 390 m a.s.l. and annual mean temperature of 18 °C. The southern limit is 18°18′N, 108°18′E with an elevation of 55 m a.s.l. and annual mean temperature of 24 °C.

It requires high nutrient and water supply during its growth and sustained maintenance and management effort from the plantation, which set limits on its areal distribution. The distribution of eucalyptus plantations in China is affected by mean annual temperature, precipitation, and elevation. In China, Eucalyptus plantations are mostly distributed in areas with low elevation (below 300 m a.s.l.), mean annual temperature of 19–21 °C, and mean annual precipitation of 1400–1600 mm.

Eucalyptus is one of the fast-growing tree species characterized by high productivity, high competitiveness, and short growth cycle. It has lacks companion tree species. It is the most important tree species for timber production. Eucalyptus provides to a large amount of timbers with relatively small area. The 9th NFI data suggests that although eucalyptus plantations account for 6.85% and 6.37% of total plantation area and stock volume in China, respectively, they contribute to logged timbers with annual cut of 3327.58 × 10^4^ m^3^, which is 17.96% of net annual cut of all timbers in China. It is indicde that eucalyptus to be an important tree species for timber logging, and it plays an irreplaceable role in the protection of natural forests in China.

However, the ecological problems caused by eucalyptus plantation management, such as land degradation, needs urgent attention^39,40^. From the analysis of soil physical and chemical properties in eucalyptus plantations, it can be seen that the decline in soil fertility caused by eucalyptus plantations is mostly caused by medium to high succession rotation regimes planting, with more than 80% of eucalyptus plantations in China being planted in short succession cycles, with shorter rotation periods than in other countries. It is generally 5–7 years, or even 3–5 years^7,21^. Compared with *Pinus massoniana* Lamb. and *Cunninghamia lanceolata* Lamb., Eucalyptus plantations can reduce soil bulk, increase the stability of water-stable aggregates, reduce soil damage, reduce soil erosion and increase soil organic matter content^41–43^. Eucalyptus plantations have a high capacity for soil carbon sequestration, peaking at 10 years for plantations over 7 years and 5 years for plantations over 7 years^38,44–46^. Eucalyptus plantations are mainly used to provide timber, and when considering land consumption and water resources, they need to be planted at low generation, mixed with multiple species, and at reduced planting densities, and planted in areas with more than 1200 mm of precipitation, the harvesting period should be extended, for example, by harvesting at the highest soil organic carbon content (5 years or 10 years). These views are basically consistent with the findings of Tang jian, Zhou Runhu and Zheng Zhouxiang et al.^32,39,40^.

This study provides valuable data and insights to character rise the growth of eucalyptus (traits) and is expected to improving the overall productivity, management and land use efficiency of eucalyptus plantations. However, there are limitations in this paper, as NFI data is used, which is surveyed every 5 years. It and lacks more precise annual monitoring data to further more finely monitor changes in eucalyptus plantations, as well as being influenced by factors such as harvesting management policies and the drive for economic interest, which could be considered for inclusion in the study if relevant data is collected in the future.

## Conclusions

1. Our study suggests that the total area of eucalyptus plantations has increased consistently in China. From 1973 to 2018, their distribution expanded from Guangxi and Guangdong Provinces to their east, north, and west, reaching Sichuan and Hunan Provinces as well as other nine provinces (districts). The total area of eucalyptus plantations in China is 546.74 × 10^4^ hectares in 2018, which is 23.77 times greater than that in 1973. The stock volume in 2018 is 21,562.90 × 10^4^ m^3^, which is 56.96 times greater than that in 1973. The total carbon storage of eucalyptus increases from 2.40 to 133.00 TgC. Its carbon density increases from 5.22 to 12.16 MgC per square hectometer, about 2.33 times of increase. Eucalyptus has become a dominant tree species for timber production in southern China. Especially during 2004–2018, the development of eucalyptus plantations gained significant attentions, and their total area, distribution, and stock volume increased rapidly along with the improvement in plantation management. It has short logging/rotation period, and is capable of storing and fixing carbon with efficiency.
2. Eucalyptus has relatively great productivity. Mean annual productivity of eucalyptus ranges from 4.14–8.57 m^3^ hm^−2^ a^−1^. Newly-cultivated lands tend to have a greater productivity, reaching 9.32–10.88 m^3^ hm^−^2 a^−1^ in some cases (Table 3). It has a mean annual carbon fixation of 5.29 t hm^−2^ a^−1^ in 2014–2018, which is more than 2 times greater than other species (*Pinus massoniana *Lamb and *Cunninghamia lanceolata *Lamb) widely distributed in the same area. The results suggest that eucalyptus is better at carbon fixing than other tree species, and is important to the global carbon balance. After 10–15 years of cultivation, the productivity of eucalyptus reaches its peak, and then starts to decrease gradually. Statistical model is established (based on 55 sample plots and their productivity variation for a time span of 25 years) to characterize how the productivity changes with time, and suggests that the productivity of eucalyptus reaches its minimum value after growing eucalyptus for 50 years. The management of eucalyptus plantations should take this into account to maintain the sustainability and soil fertility of the corresponding lands.
3. Eucalyptus plantations can be abandoned and used for other purposes after certain period of time, and the abandonment rate is high despite their rapid expansion in China. A quarter of the eucalyptus plantations will stop growing eucalyptus after 5 years of plantation operation. Based on the 5th to 9th NFIs data, there are 561 sample plots abandoned (regardless of when they were planted) among a total of 1843 plots (abandonment rate: 30.44%). The abandonment rate reaches its peak (32%) after 15 years of growing eucalyptus. The cumulative abandonment rates are about 50% and 75% after 10 and 20 years of plantation operation. In general, all plantations would be used for other purposes after 50 years of growing eucalyptus. Eucalyptus consumes soil fertility fast, and requires sufficient supply of water. So far, only 20% of the abandoned plantations have replanted eucalyptus. There is a potential to increasing in the replanting rate, and reducing in the time window from not growing eucalyptus to replanting it. Using more scientific and quantitative measures for plantation management, such as land and crop rotation, is essential to the sustainable profitability, soil fertility restoration, and land use efficiency of eucalyptus plantations.
